# Analysis of influencing factors and construction of predictive model for major gastrointestinal bleeding after receiving VTE prophylactic anticoagulants in non-tumor patients

**DOI:** 10.3389/fmed.2025.1618626

**Published:** 2025-08-11

**Authors:** Xiaoxue Fu, Huiya Ren, Yingbo Yang, Zhixue Niu, Linjie Zhang

**Affiliations:** ^1^Baoding First Central Hospital, Baoding, Hebei, China; ^2^Baoding First Hospital, Baoding, Hebei, China

**Keywords:** Padua risk assessment model, improve-bleed risk assessment model, venous thromboembolism, major gastrointestinal bleeding, prediction model

## Abstract

**Objective:**

This study explored the factors associated with major gastrointestinal bleeding (MGB) after the use of prophylactic anticoagulants in patients with high risk of venous thromboembolism (VTE) and developed a predictive model to more accurately identify patients at high risk of MGB.

**Methods:**

We collected related data on patients with a high risk of VTE and a low risk of bleeding between January 2020 and December 2024. Propensity score matching (PSM) was conducted to balance the important factors related to the disease status between the non-MGB and MGB groups. By applying univariate and multivariable logistic regression, predictive factors were refined to construct the nomogram. The prediction accuracy of the model was assessed through bootstrapping method (1,000 bootstrap samples), Hosmer-Lemeshow test, and calibration curve analysis. Decision curve analysis (DCA) was also used to assess the clinical applicability of the model.

**Results:**

In total, 1,206 eligible patients (1,060 non-MGB vs. 146 MGB) were included. After PSM, the non-MGB group (*n* = 146) and the MGB group (*n* = 146) were more balanced in baseline variables. The results of the multivariable analysis showed that hemoglobin level (OR = 0.883, 95% CI: 0.855–0.911, *p* < 0.001), albumin level (OR = 0.834, 95%CI: 0.754–0.922, *p* < 0.001) and use of proton-pump inhibitors (PPIs) or H_2_-receptor antagonists (H_2_RAs) (OR = 0.375, 95%CI: 0.167–0.843, *p* = 0.018) were associated with a reduced risk of MGB. The congruence of the model’s predictions with actual findings was confirmed by the calibration curve. Furthermore, DCA affirmed the clinical value of the model in predicting MGB.

**Conclusion:**

The addition of anemia and hypoproteinemia to the Improve-Bleed risk assessment model may help the timely and accurate screening of patients at high risk of MGB. The use of PPIs or H_2_RAs was associated with a reduced risk of MGB in patients receiving prophylactic anticoagulants for VTE.

## 1 Introduction

Venous thromboembolism (VTE) constitutes a group of thromboembolic diseases, including pulmonary thromboembolism and deep vein thrombosis. In 2008, a global study on the prevention status of VTE in hospitalized patients found that 52% of hospitalized patients were at risk of VTE (1), while up to 900,000 patients experienced VTE for the first time during hospitalization (2). Approximately 50% of patients with proximal deep vein thrombosis can progress to pulmonary embolism, and 79% of patients with pulmonary embolism have lower extremity deep vein thrombosis (3). Nearly 21% of cases of pulmonary embolism are fatal, resulting in approximately 40,000 annual deaths in the United States, and 75% of fatal VTE occur in hospitalized patients (4). The 9th edition of the Guidelines for Antithrombotic Treatment and Prevention published by the American Association of Chest Physicians in 2012 recommends the Padua risk assessment model for VTE risk screening in hospitalized patients (5). To date, the Padua risk assessment model (Table 1) has been considered the best model for assessing VTE risk in the field of internal medicine. Evidence-based guidelines have shown that drug prevention can reduce the risk of PE by 57% (6). However, in many older patients with acute conditions and multiple complications, clinicians have to consider the risk of major bleeding when initiating pharmacoprophylaxis for VTE. The clinical application of medications for VTE prevention is still not ideal because of the heterogeneity in the definition of high-risk patients and clinicians’ concerns about major bleeding.

**Table 1:** Padua risk assessment model (high risk of VTE ≥ 4).

Baseline features	Points
Active cancer^*^	3
Previous VTE (with the exclusion of superficial vein thrombosis)	3
Reduced mobility. Bed rest with bathroom privileges (either due to patients’ limitations or on physician order) for at least 3 days.	3
Already known thrombophilia. Carriage of defects of anti-thrombin, protein C or S, factor V Leiden, G20210A prothrombin mutation, anti-phospholipid syndrome.	3
Recent (≤1 month) trauma and/or surgery	2
Elderly age (≥70 years)	1
Heart and/or respiratory failure	1
Acute myocardial infarction or ischemic stroke	1
Acute infection and/or rheumatologic disorder	1
Obesity (BMI ≥ 30)	1
Ongoing hormonal treatment	1

^*^Patients with local or distant metastases and/or in whom chemo-therapy or radiotherapy was conducted in the last 6 months.

Gastrointestinal bleeding (GIB) accounts for 40% of major anticoagulant-related bleeding events and serves as an independent risk factor for poor outcomes (7). The highest incidence of major bleeding can be observed at the beginning of anticoagulant therapy (8). The Improve-Bleed risk assessment model is the most commonly used model for assessing hemorrhage risk in patients receiving VTE prophylaxis. However, in clinical practice, a proportion of the patients with low bleeding risk were found to suffer from major gastrointestinal bleeding (MGB) while receiving VTE prophylactic anticoagulants. Thus, it is of great significance for clinicians to identify high-risk groups of MGB accurately. Therefore, this study mainly explored the factors associated with MGB caused by prophylactic anticoagulants in patients with a high risk of VTE and a low risk of bleeding.

## 2 Study subjects and methods

### 2.1 Study subjects

In total, 4,328 patients who were admitted to the Baoding First Central Hospital from January 2020 to December 2024 were selected for this study. The participants exhibited high risk in the Padua risk assessment model (Table 1) and low risk in the Improve-Bleed risk assessment model (Table 2) (9, 10). All patients who meet the criteria for anticoagulation therapy are informed of its benefits and risks and are required to sign an informed consent form. However, 614 patients have declined anticoagulant therapy. The remaining 3,714 received prophylactic anticoagulants [including low molecular weight heparin (LMWH), enoxaparin, and nadheparin]. Among these cases, we observed 114 cases of skin bruising/mucosal bleeding, 92 genitourinary bleeding events, and 7 intracranial hemorrhage (ICH), with most being mild. Two independent internists retrospectively reviewed the medical records of patients, and patients who met the following criteria were excluded: (1) long-term use of anticoagulants; (2) history of cancer; (3) hospitalization for less than 5 days; (4) VTE was diagnosed during hospitalization; (5) experiencing any bleeding event other than MGB; (6) lack of some critical clinical data. Based on the above exclusion criteria, 1,206 patients were finally enrolled.

**Table 2:** Improve-bleed risk assessment model.

Bleeding risk factor	Points
Active gastric or duodenal ulcer	4.5
Prior bleeding within the last 3 months	4
Thrombocytopenia (<50 × 10^9^/L)	4
Age ≥85 years	3.5
Liver failure (INR > 1.5)	2.5
Severe kidney failure (GFR < 30 mL/min/m^2^)	2.5
Admission to the ICU/CCU	2.5
Central venous catheter	2
Rheumatic disease	2
Active malignancy	2
Age: 40–84 years old	1.5
Male	1
Moderate kidney failure (GFR: 30–59 mL/min/m^2^)	1

INR: international normalization ratio, GFR: glomerular filtration rate, ICU: intensive care unit, CCU: coronary care unit.

Low risk: score < 7.

High risk: score ≥ 7.

### 2.2 Methods

#### 2.2.1 Diagnosis of MGB

Diagnostic criteria for MGB were as follows: (1) as defined by the International Society of Thrombosis and Hemostasis (ISTH): bleeding leading to a decrease in hemoglobin level ≥ 2 g/dL or leading to the transfusion of ≥ 2 units of whole blood or red cells (11); (2) presence of GIB signs, such as hematemesis and/or hematochezia.

#### 2.2.2 Clinical data collection

**Figure 1:**
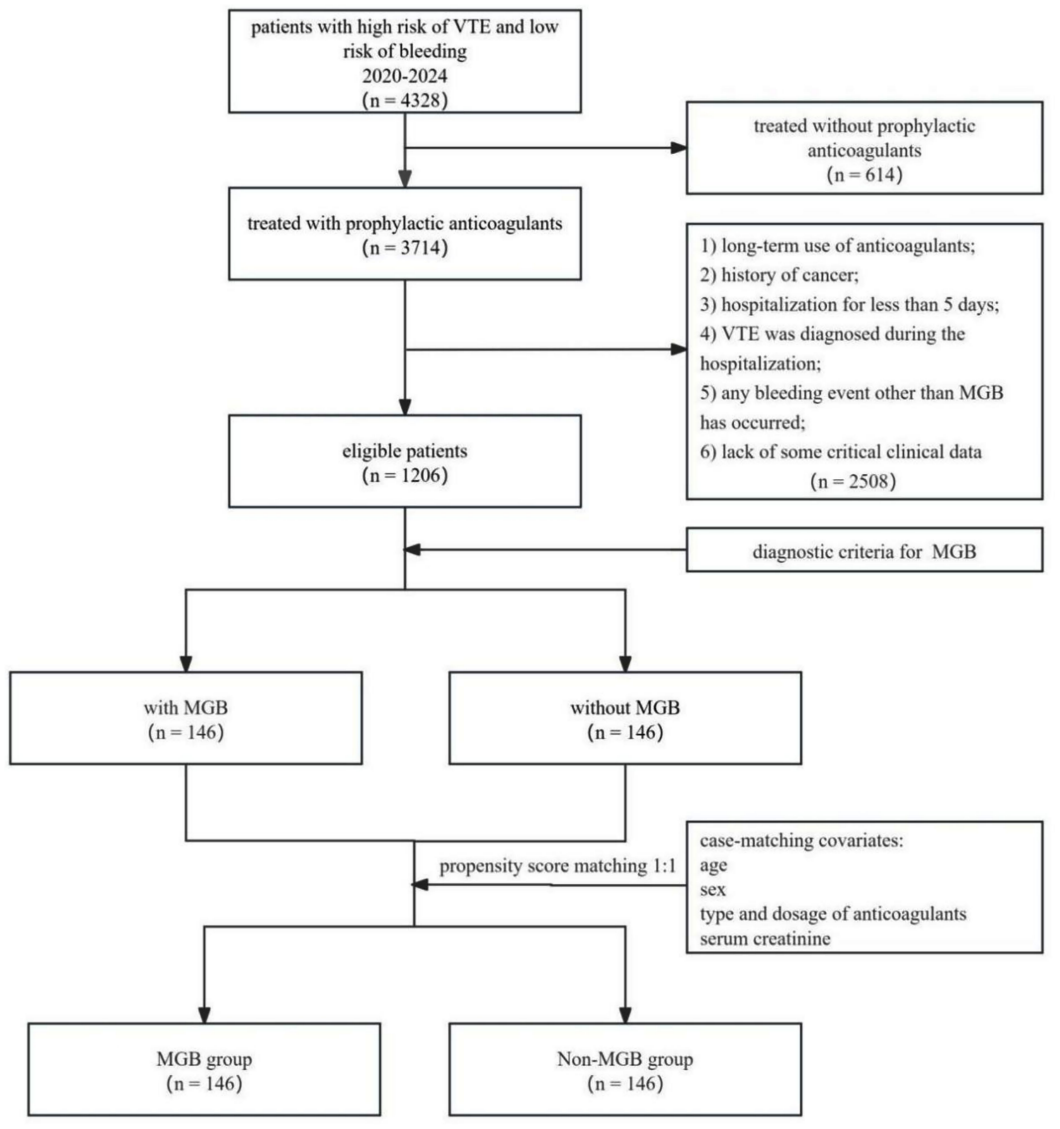
Study flow diagram. VTE, Venous thromboembolism; MGB, major gastrointestinal bleeding.

Based on the presence of concomitant MGB, patients were classified into two groups: those with MGB (*n* = 146) and those without MGB (*n* = 1,060). After propensity score matching (PSM) with four covariates (age, sex, type and dosage of anticoagulantsand, and serum creatinine) using parameters of “method = ‘nearest neighbour’, ratio = 1:1, caliper = 0.01,” 146 patients without MGB (non-MGB group) were matched to 146 patients with MGB (MGB group) (Figure 1).

Data on the following variables were collected from 292 patients: age, sex, type of anticoagulant, medication, use of antiplatelet agents, use of PPIs or H_2_RAs, history of underlying conditions (e.g., active gastric or duodenal ulcer, thrombocytopenia, liver failure, prior bleeding within the last 3 months, moderate or severe kidney failure, and rheumatic disease), laboratory data [e.g., platelet count, hemoglobin level, international normalized ratio (INR), D-dimer, serum albumin, high-density lipoprotein (HDL), low-density lipoprotein (LDL), C-reactive protein (CRP), procalcitonin (PCT), serum creatinine, pro-B-type natriuretic peptide (Pro-BNP)], and the Improve-Bleed risk score within 24 h of hospital admission.

The study complied with the principles of the Declaration of Helsinki. This study was approved by the Ethics Committee of Baoding First Central Hospital.

### 2.3 Statistical analysis

PSM with four covariates (age, sex, type and dosage of anticoagulants, and serum creatinine) using the parameter of “method = ‘nearest neighbour’, ratio = 1:1, caliper = 0.01” was conducted using R version 4.3.1. The Shapiro–Wilk test was used to investigate whether continuous variables conformed to normal distribution. For variables with normal distribution in both groups, the difference between groups was expressed as mean ± SD. The difference in non-normally distributed variables was assessed using the Mann–Whitney U test and expressed as median (25th, 75th). Dichotomous variables were compared using a 2 × 2 contingency table and composition ratios were compared using the chi-square test. Variables were considered potential risk factors if *p*-values were < 0.05 in an unadjusted univariate analysis. These variables were then entered into a multivariable logistic regression analysis. Risk variables in the final model were considered to be significant contributors to bleeding if their *p*-values were < 0.05. Statistical analyses were conducted using SPSS 26.0 (IBM, New York, USA).

A nomogram model was developed using R software. The bootstrapping method (1,000 bootstrap samples) was conducted to prevent over-fitting of the model and validate its stability. We also plotted the receiver operating characteristic (ROC) curve, calculated the area under the curve (AUC), and determined the optimal cutoff value using Youden’s index to maximize the sensitivity and specificity of the model (12). Calibration plots and the Hosmer-Lemeshow test were employed to confirm the accuracy of the prediction model. Additionally, decision curve analysis (DCA) was used to assess the clinical benefit of the prediction model (13). Data were processed and analyzed using R version 4.3.1 software.

## 3 Results

### 3.1 Patient characteristics

The clinical characteristics of 292 eligible patients are shown in Table 3. Among patients receiving prophylactic anticoagulants, 146 experienced MGB during hospitalization. After PSM, the non-MGB group (*n* = 146) and the MGB group (*n* = 146) were more balanced in terms of baseline variables. Notably, the difference in age and sex was addressed and the most important variables (i.e., anticoagulant type and dosage, and serum creatinine) were more comparable between the two groups.

**Table 3:** Demographics of patients receiving prophylactic anticoagulants.

Variable	Non-MGB group (*n* = 146)	MGB group (*n* = 146)	Statistic	*p*
Sex [*n*(%)]			–	–
Male	72 (49.3)	72 (49.3)		
Female	74 (50.7)	74 (50.7)		
Type of anticoagulant medication [*n*(%)]			–	–
Enoxaparin	72 (49.3)	72 (49.3)		
Nadroparin	55 (37.7)	55 (37.7)		
Low molecular weight heparin	19 (13.0)	19 (13.0)		
Age (year)[*x* ± $\bar{x}$ ]	79.6 ± 8.3	78.4 ± 9.3	2.042	0.154
Serum creatinine [μmol/L, M(25th,75th)]	78.0 (58.4,113.5)	74.5 (56.6,120.6)	−0.442	0.658
Antiplatelet agent use [*n*(%)]	48 (32.9)	44 (30.1)	0.254	0.614
PPIs or H_2_RAs use [*n*(%)]	113 (77.4)	70 (48.0)	27.067	<0.001
Liver failure (INR > 1.5) [*n*(%)]	4 (2.7)	4 (2.7)	–	–
Prior bleeding within the last 3 months [*n*(%)]	5 (3.4)	4 (2.7)	0.115	0.735
Central venous catheter [*n*(%)]	20 (13.7)	23 (15.8)	0.245	0.620
Rheumatic disease [*n*(%)]	3 (2.1)	5 (3.4)	0.514	0.473

*p* values are calculated using student t or χ^2^ tests. PPIs, Proton-pump inhibitors; H_2_RAs, H_2_-receptor antagonists; INR, International normalized ratio; GFR, glomerular filtration rate.

Rheumatic disease: encompasses a broad spectrum of musculoskeletal, arthritic and connective tissue disorders, including rheumatoid arthritis (RA), osteoarthritis (OA), fibromyalgia, gout, vasculitides, systemic sclerosis, systemic lupus erythematosus (SLE), ankylosing spondylitis and psoriatic arthritis, etc.

There were no significant differences between the two groups in terms of the use of antiplatelet agents, active gastric or duodenal ulcer, thrombocytopenia, liver failure, prior bleeding within the last 3 months, moderate or severe kidney failure, central venous catheter insertion, and rheumatic disease between (*p* > 0.05).

Patients receiving proton-pump inhibitors (PPIs)/H_2_-receptor antagonists (H_2_RAs) during hospitalization were at lower risk of MGB. The difference was statistically significant (*p* < 0.05).

### 3.2 Univariate analysis

Univariate analysis (Table 4) showed that patients with lower hemoglobin, albumin, LDL, or HDL levels were at a higher risk of MGB.

**Table 4:** Potential factors of patients receiving prophylactic anticoagulants.

Variables	Non-MGB group (*n* = 146)	MGB group (*n* = 146)	Statistic	*p*
Leucocyte count [×10^9^/L, M(25th,75th)]	7.27 (5.53, 9.46)	7.21 (4.90, 9.96)	−0.502	0.616
Platelet count [×10^9^/L, M(25th,75th)]	212 (168, 267)	239 (167, 299)	−1.785	0.074
Hemoglobin [g/L, *x* ± $\bar{x}$ ]	119.06 ± 16.65	87.58 ± 13.44	7.291	0.007
INR [M(25th,75th)]	1.17 (1.10, 1.27)	1.18 (1.10, 1.26)	−0.102	0.919
D-dimer [mg/L, M(25th,75th)]	2.26 (1.25, 3.43)	2.27 (1.19, 3.90)	−0.314	0.754
Serum albumin [g/L, *x* ± $\bar{x}$ ]	33.31 ± 5.08	28.16 ± 3.70	13.776	<0.001
HDL [mmol/L, M(25th,75th)]	1.02 (0.85, 1.21)	0.98 (0.82, 1.12)	−2.096	0.036
LDL [mmol/L, M(25th,75th)]	2.09 (1.59, 2.61)	1.85 (1.51, 2.21)	−2.776	0.005
CRP[mg/L, M(25th,75th)]	66.20 (22.00, 104.28)	64.90 (22.13, 104.18)	−0.264	0.792
PCT [ng/mL, M(25th,75th)]	0.33 (0.10, 0.72)	0.38 (0.12, 1.18)	−1.387	0.165
Pro-BNP [pg/mL, M(25th,75th)]	1653.00 (693.50, 4463.00)	1536.50 (636.50, 4522.75)	−0.446	0.656
Padua risk score [M(25th,75th)]	5.00 (4.00, 5.25)	5.00 (4.00, 6.00)	−0.157	0.875
Improve-Bleed risk score [M(25th,75th)]	3.75 (2.50, 4.63)	3.50 (2.50, 4.50)	−0.282	0.776

*p* values are calculated using Student *t* or Z tests.

INR, International normalized ratio; HDL, High density lipoprotein; LDL, Low density lipoprotein; CRP, C-reactive protein; PCT, Procalcitonin; Pro-BNP, Pro-B-type natriuretic peptide.

### 3.3 Multivariable analysis

Multiple logistic regression analysis of different variables (Table 5) (*p* < 0.05) was used to screen independent risk factors. The results of the multivariable analysis showed that hemoglobin level (OR = 0.883, 95%CI: 0.855–0.911, *p* < 0.001) and albumin level (OR = 0.834, 95%CI: 0.754–0.922, *p* < 0.001) were associated with a decreased risk of MGB secondary to anticoagulation therapy. Furthermore, using PPIs or H_2_RAs (OR = 0.375, 95%CI: 0.167–0.843, *p* = 0.018) was associated with a decreased risk of MGB during prophylactic anticoagulant therapy.

**Table 5:** Multivariable analysis of potential factors for MGB in patients receiving prophylactic anticoagulants.

Variables	OR (95% CI)	*p*
PPIs or H_2_RAs use	0.375 (0.167–0.843)	0.018
Hemoglobin	0.883 (0.855–0.911)	<0.001
Serum albumin	0.834 (0.754–0.922)	<0.001

OR, odds ratio; 95% CI, 95% confidence interval; PPIs, Proton-pump inhibitors; H_2_RAs, H_2_-receptor antagonists.

### 3.4 Development of the prediction models

**Figure 2:**
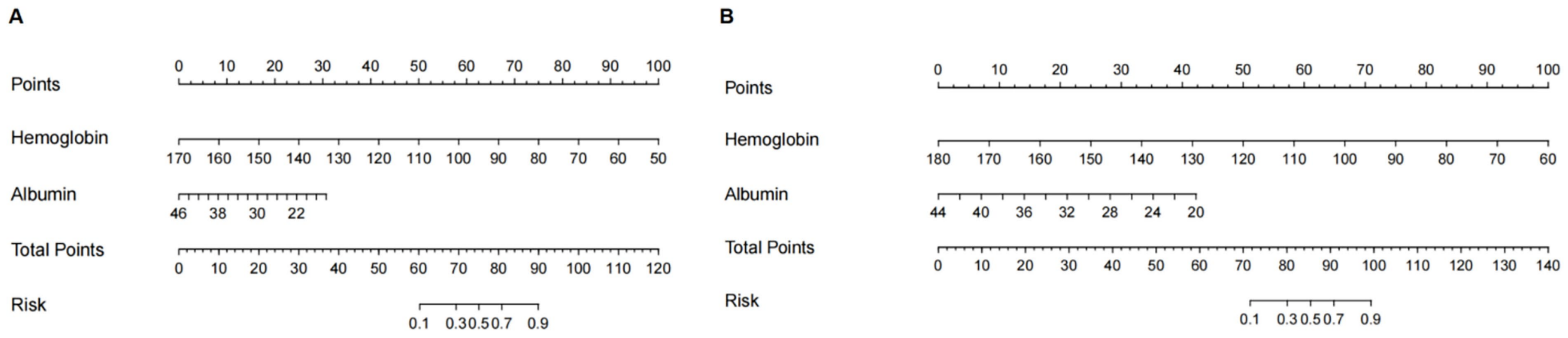
The nomogram models for quantifying individual risk of MGB after receiving prophylactic anticoagulants. **(A)** Use of PPIs/H_2_RAs (Model 1). **(B)** Non-use of PPIs/H_2_RAs (Model 2).

Using R software, nomogram models were developed to predict the risk of MGB after receiving prophylactic anticoagulants, incorporating the two influencing factors identified through multivariable analysis. Summing these scores yielded an overall score, which was then used to estimate the risk of MGB. An elevated total score indicated an increased risk of MGB. Given that the use of PPIs/H_2_RAs is a modifiable intervention rather than a fixed baseline characteristic, we established predictive models, respectively, for the use or non-use of PPIs/H_2_RAs patients (see Figure 2).

### 3.5 Evaluation of the prediction models

**Figure 3:**
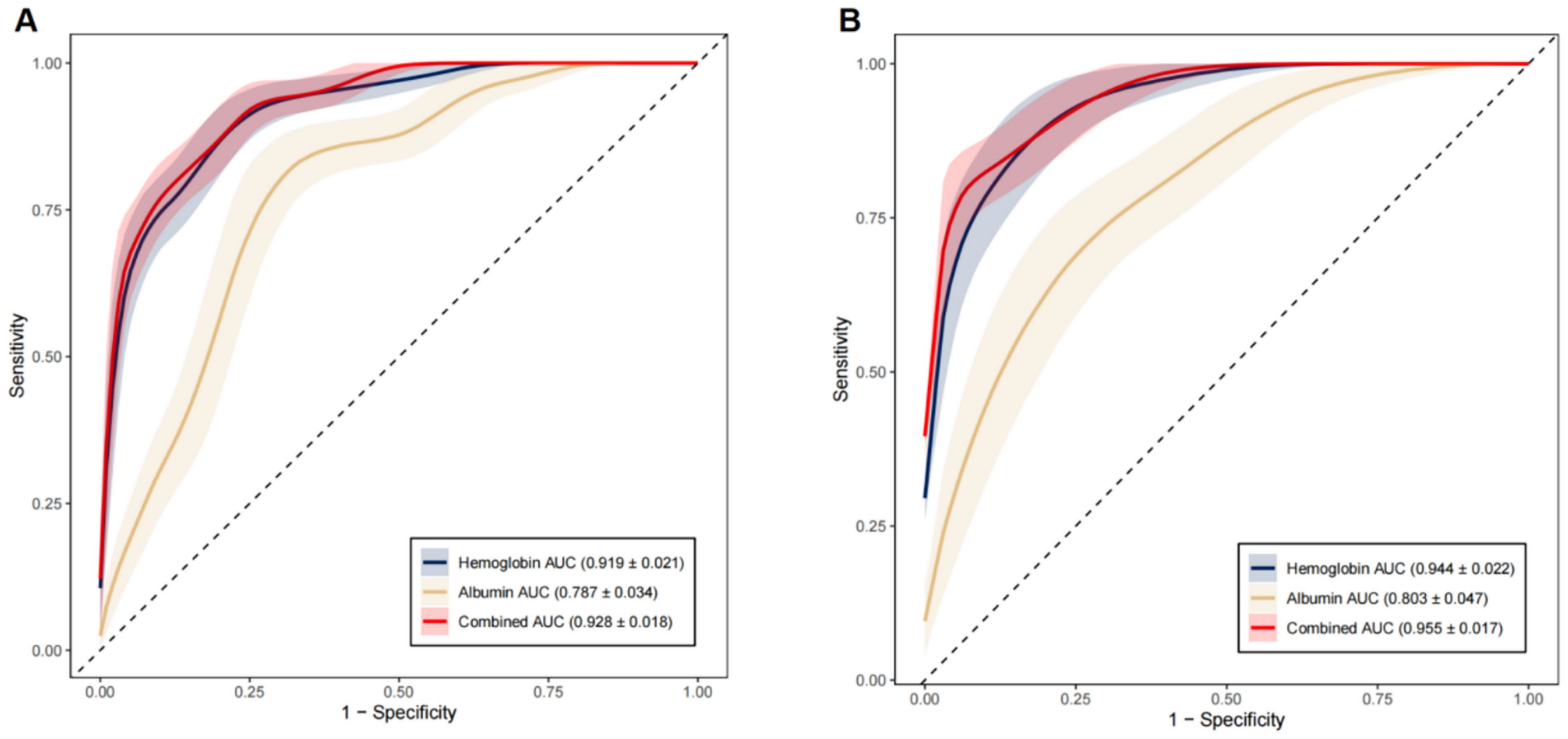
Area under the ROC curve for models to predict MGB and individual predictors. **(A)** Use of PPIs/H_2_RAs (Model 1). **(B)** Non-use of PPIs/H_2_RAs (Model 2). MGB, major gastrointestinal bleeding; ROC, receiver operating characteristic; AUC, area under the curve.

The bootstrapping method (1,000 bootstrap samples) was conducted for internal validation to confirm the model stability. The prediction Model 1 (AUC = 0.928, 95% CI: 0.910 to 0.946) and Model 2 (AUC = 0.955, 95% CI: 0.938 to 0.972) were both found to have good discrimination accuracy, supporting the excellent discriminatory ability of these models. The contribution of each included predictor for each model is shown in Figure 3.

ROC curve analysis of two statistically significant continuous variables, namely hemoglobin and albumin levels on admission, showed that the cutoff values of hemoglobin and albumin levels in Model 1 were 104.16 g/L and 30.93 g/L and in Model 2100.11 g/L and 30.32 g/L. The incidence of MGB was significantly higher in patients with hemoglobin levels < 104.16 g/L and albumin levels < 30.93 g/L who were using PPIs/H_2_RAs. The incidence of MGB was significantly higher in patients with hemoglobin levels < 100.11 g/L and albumin levels < 30.32 g/L who were not using PPIs/H_2_RAs (Table 6).

**Table 6:** Values of hemoglobin and albumin for risk assessment of secondary MGB.

Variables	Cutoff value	Sensitivity	Specificity	Youden’s index
Hemoglobin^a^ (g/L)	104.16	0.873	0.837	0.711
serum albumin^a^ (g/L)	30.93	0.830	0.712	0.543
Hemoglobin^b^ (g/L)	100.11	0.883	0.877	0.760
serum albumin^b^ (g/L)	30.32	0.745	0.771	0.516

a In the model 1.

b In the model 2.

MGB, major gastrointestinal bleeding; AUC, area under the curve.

**Figure 4:**
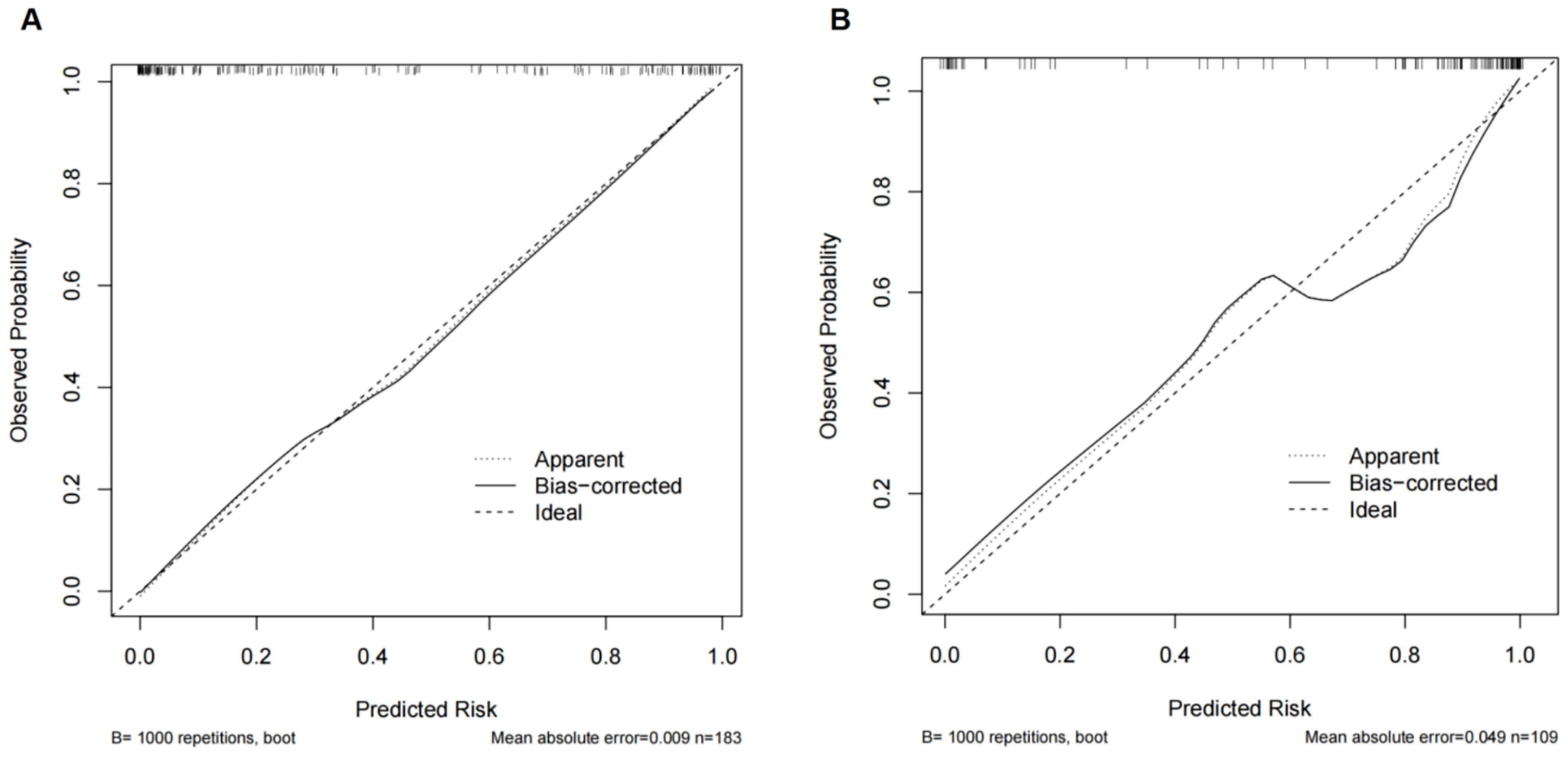
Evaluation of prediction models for predicting the risk of secondary MGB after receiving prophylactic anticoagulants. **(A)** Use of PPIs/H_2_RAs (Model 1). **(B)** Non-use of PPIs/H_2_RAs (Model 2).

The Hosmer-Lemeshow test for Model 1 calibration gave a χ^2^ = 9.279 and a *p* value of 0.319, for Model 2 calibration gave a χ^2^ = 7.514 and a *p* value of 0.482 (Figure 4), suggesting no significant difference between the observed and predicted probabilities of MGB. These findings suggest that the model does not misinterpret the data.

### 3.6 Clinical utility of the prediction models

**Figure 5:**
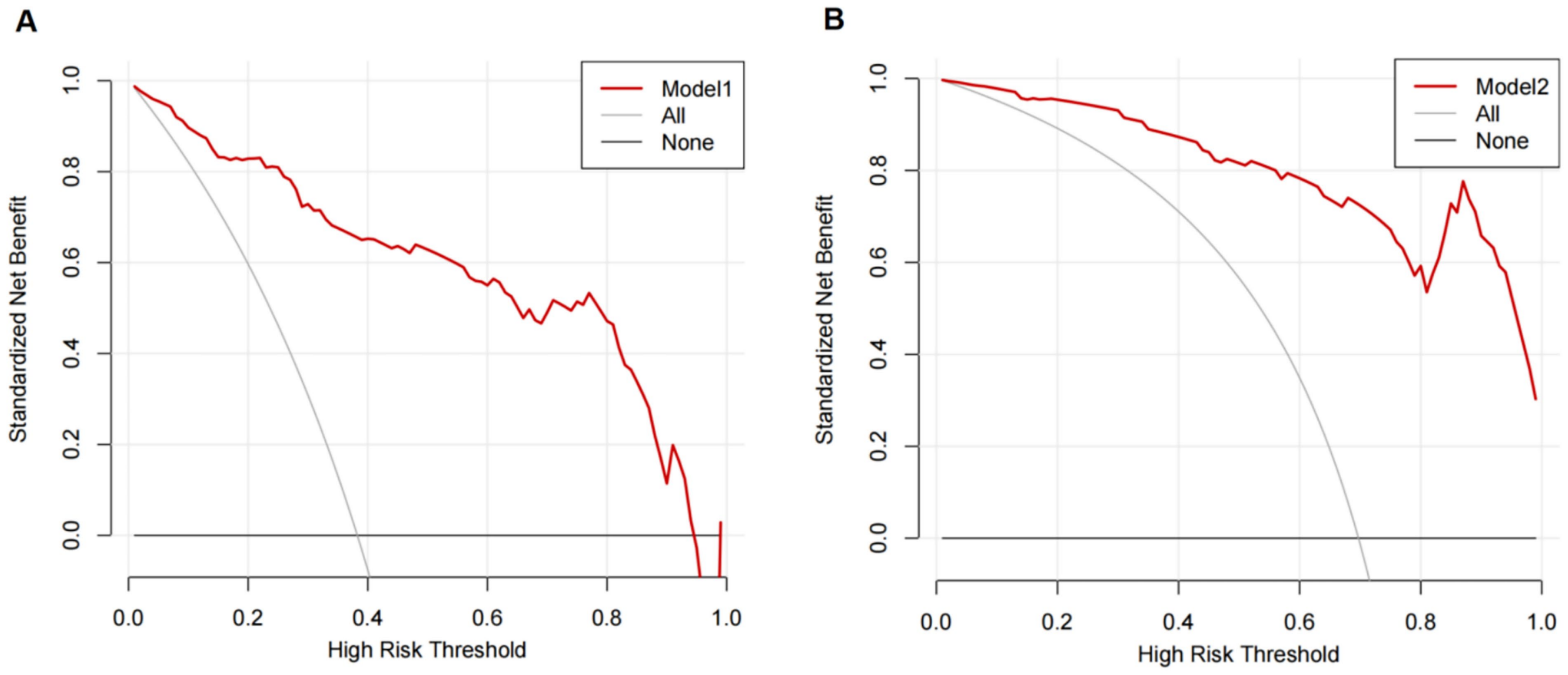
Decision curves of the predictive model for the risk of secondary MGB after receiving prophylactic anticoagulants. **(A)** Use of PPIs/H_2_RAs (Model 1). **(B)** Non-use of PPIs/H_2_RAs (Model 2).

The graph shows that this model shows a high clinical application value (Figure 5).

## 4 Discussion

VTE is a systemic disease affected by genetic, environmental, and behavioral factors. Hospitalization alone is the most important risk factor for VTE (14) because hospitalized patients are more likely to have obesity, old age, poor mobility, and active inflammation due to potential comorbidities and acute diseases (e.g., sepsis and shock) (15). Due to its high incidence, dangerous condition, and lack of typical clinical manifestations, VTE is prone to misdiagnosis and missed diagnosis, increasing the risk of unexpected death among internal medicine inpatients (16). Studies recommend that medical patients with a Padua prediction score ≥ 4 should receive pharmacological prophylaxis during hospitalization as long as their Improve-Bleed risk score < 7. Those with a bleeding risk score of 7 or more may benefit from mechanical prophylaxis until the bleeding risk is reduced (17).

However, in clinical practice, some patients with low bleeding risk develop MGB after the use of prophylactic anticoagulants. Therefore, we focused on patients with MGB after receiving prophylactic anticoagulants. This study found that hemoglobin levels and serum albumin levels were associated with a reduced risk of MGB after receiving prophylactic anticoagulants. Furthermore, the use of PPIs or H2RAs protected against MGB.

Hemoglobin level (OR = 0.883, 95%CI: 0.855–0.911, *p* < 0.001) was associated with decreased risk of MGB. A study showed that low hemoglobin levels (OR = 4.8, 95%CI: 1.5–16.0) were associated with an increased risk of gastrointestinal bleeding in patients with gastrointestinal cancer receiving edoxaban (18). A meta-analysis with 34 studies showed that GIB subsequent to the use of anticoagulants may be associated with a history of bleeding (OR = 3.26, 95%CI: 1.86–5.73) (19) and anemia (OR = 1.48, 95%CI: 1.10–1.98) (20, 21). It has also been shown that anemia independently predicts the risk of GIB (22). The thresholds of anemia are different (<13, 12, or 10 g/dL) in common bleeding risk scores, such as HAS-Bled (23), OBRI (24), Kearon (25), Shireman (26), RIETE (27), HEMORR2HAGES (28), ATRIA (29), and ORBIT (30), but all of them emphasize the value of anemia. A study evaluated the risk of bleeding among patients with anemia and atrial fibrillation taking direct oral anticoagulants. The standardized absolute 1-year risk difference for composite bleeding increased by 0.96% for patients with moderate or severe anemia compared to patients without anemia. This risk was mainly driven by increased standardized absolute 1-year risk for serious GI bleeding (31). Anemia, to some extent, indicates that occult GIB may have started before receiving prophylactic anticoagulants.

Albumin is an indicator reflecting the nutritional status of protein. In this study, the albumin level (OR = 0.834, 95%CI: 0.754–0.922, *p* < 0.001) was associated with reducing the occurrence of MGB in patients receiving prophylactic anticoagulants. By analyzing albumin levels and mortality among 5,894 patients with acute conditions, Jellinge ME *et al.* identified hypoproteinemia as a strong predictor of 3-day all-cause mortality (32). Hypoproteinemia may be associated with various factors, such as malignancy, severe malnutrition, kidney disease, chronic liver disease, and infection. Severe malnutrition may weaken the protection mechanism of the gastric mucosa, decelerate the repair process, and make patients prone to bleeding from the digestive tract.

This study found that the combined application of PPIs/H_2_RAs reduced the occurrence of MGB subsequent to the use of anticoagulants. In a trial with 3,761 outpatients receiving dual antiplatelet therapy, PPIs reduced the risk of GIB (33). A propensity-matched study with 37,966 hospitalized patients showed that PPIs were associated with a reduced risk of clinically significant bleeding after adjusting for confounding factors (OR = 0.58, 95% CI: 0.37–0.91, *p* < 0.001) (34). A meta-analysis that enrolled 9,533 patients showed that PPIs were associated with a reduced risk of clinically important upper gastrointestinal bleeding (RR = 0.51, 95% CI: 0.34–0.76; high-certainty evidence) (35). A systematic review and network meta-analysis with 12,660 critically ill patients in 72 trials found that both PPIs and H_2_RAs decrease the risk of clinically important bleeding (36).

There are limitations that should be considered when interpreting the results. Data were collected in a retrospective manner without randomization. We relied on data recorded by healthcare providers, and some cases may have been missed. We focused on risk prediction for MGB. In clinical practice, bleeding is often multi-focal, but our exclusion criteria may limit generalizability. In addition, the small sample size of our study limited statistical power, and the results should merely be regarded as exploratory. Therefore, more evidence and external validation in different populations are needed, and further studies are needed to develop a new scoring system covering all potential risk factors.

## 5 Conclusion

Both hemoglobin level and albumin level were associated with a reduced risk of MGB following prophylactic anticoagulation therapy for VTE. In addition, the use of PPIs/H2RAs decreased the incidence of MGB subsequent to anticoagulation therapy. Although the Improve-Bleed risk assessment model is widely recognized, incorporating anemia and hypoproteinemia into this model may facilitate the identification of patients at high risk of MGB.

## Data Availability

The raw data supporting the conclusions of this article will be made available by the authors, without undue reservation.
